# Navigating tensions between public and commercial interests: a case study of open source biosensors for detecting water contaminants in Argentina

**DOI:** 10.3389/fmed.2024.1268950

**Published:** 2024-01-11

**Authors:** Alejandro D. Nadra

**Affiliations:** Departamento de Fisiología, Biología Molecular y Celular, Facultad de Ciencias Exactas y Naturales, Instituto de Biociencias, Biotecnología y Biología Traslacional, Consejo Nacional de Investigaciones Científicas y Técnicas, Universidad de Buenos Aires, Buenos Aires, Argentina

**Keywords:** water contamination, biosensors, conflicts of interest, arsenic, public health

## Introduction

**Figure 1:**
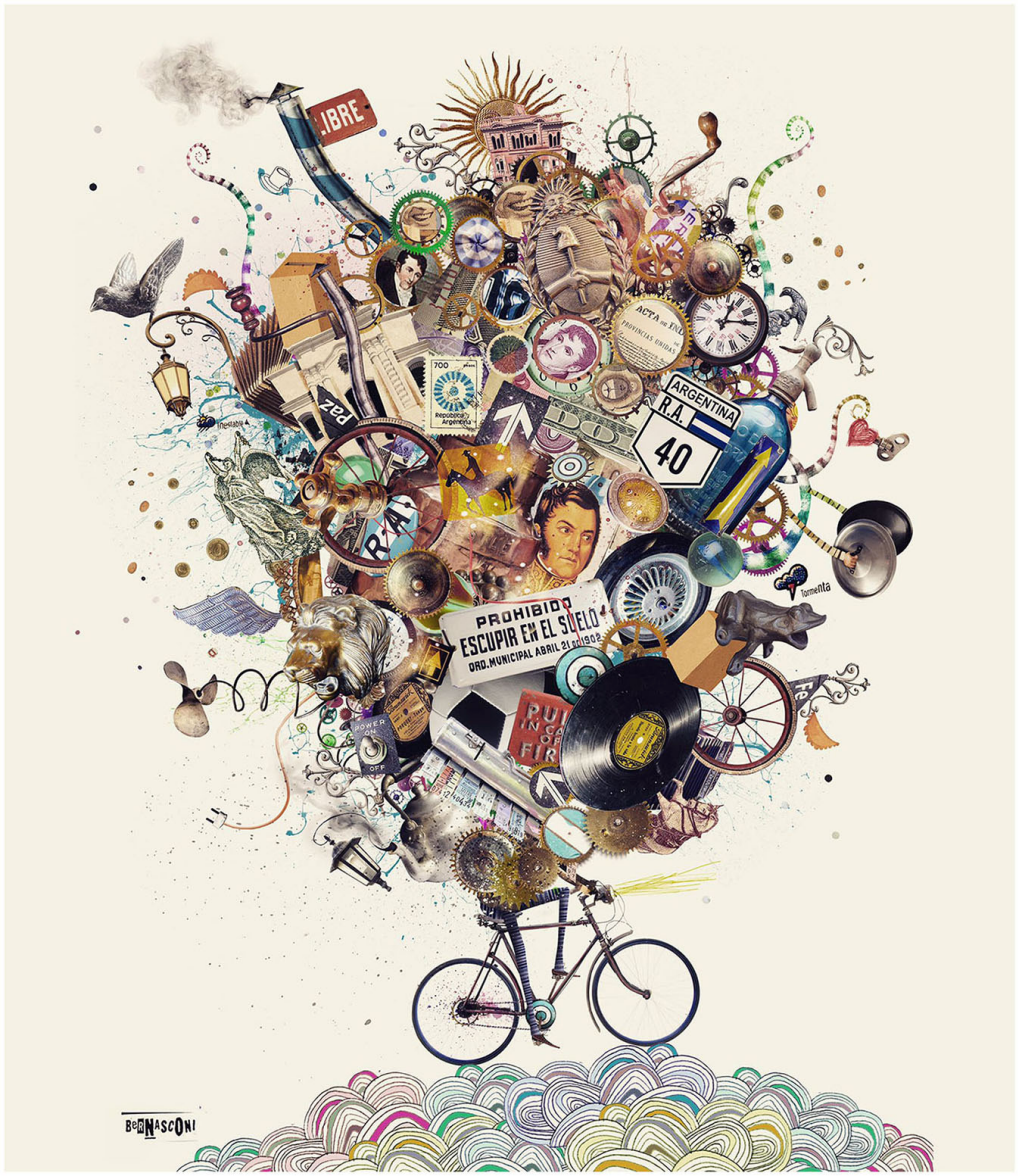
Metaphor of the many things we carry on and must deal with. An appropriate balance between them allows us to go ahead. Reproduced with permission of its author, Pablo Bernasconi.

Access to clean and safe water is a fundamental human right (1), and ensuring its quality is paramount for public health. The path to provide safe water to each individual presents tensions or conflicts that can arise due to various factors, such as: regulatory Standards (strict limits on the permissible levels of contaminants may be resisted by companies as they could potentially increase their operational costs); Economic Interests (water contamination may result in negative consequences for industries relying on clean water, such as agriculture or tourism. In such cases, commercial interests might prioritize maintaining economic activities over addressing the contamination issue, potentially conflicting with the public's interest in having clean and safe water); Liability and Responsibility (Delays in assigning responsibilities can hinder the resolution of the contamination problem and create conflicts between commercial and public interests); Access to Information (Governmental bodies and regulatory agencies may have access to scientific studies, testing results, and industry data, but they may be reluctant to disclose certain information due to potential legal consequences); Remediation Costs (its funding can create conflicts between companies that may have contaminated or should provide clean water and governments that must guarantee access to clean water). In addition, the absence of long term state policies and its concomitant sustained political support and funding as well as ineffective communication and personal egos (particular issue among scientists) contribute to a difficult journey (Figure 1).

It is impossible to cover in depth all these facets in one article. Thus, this commentary explores the challenges and controversies encountered during 10 years in the development of biosensors for detecting water contaminants in the Argentinian context (2–4). Despite the absence of malicious intent, conflicts of interest have impeded or even blocked the implementation of technological solutions to address social issues. The discrepancies between initial expectations and the harsh reality, as well as conflicting agendas among stakeholders, have hampered progress in achieving the predefined objective of improving people's lives. This paper highlights the tensions that arose during the development process and emphasizes the need for cooperation between several agents and to balance interests to overcome these hurdles, being essential the -good- intervention of governments and wise advice from supra-national organizations.

### Arsenic contamination of drinking water

Examples of drinking water scarcity around the world are alarming themselves and expose the populations to additional risks, such as arsenic poisoning. This may happen in whichever place where superficial water is not available and well groundwater is used instead. Water that may naturally contain contaminants as arsenic, one of the top 10 chemicals of public health concern for the WHO and that may be responsible for nearly 43 000 deaths annually in Bangladesh (5). Arsenic poisoning in Bangladesh, for example, has emerged as a significant health crisis stemming from the widespread use of well water. With the aim of providing a seemingly accessible and self-sufficient water source, millions of individuals in rural areas turned to shallow tube wells, unaware of the hidden danger lurking within. Tragically, these wells have become silent perpetrators of arsenic contamination during decades, leading to severe health consequences for the population (6).

In 2008, the WHO established a permissible concentration limit of 10 ppb (parts per billion) for arsenic in water intended for human consumption (7). It was mentioned that concentrations above 50 ppb are toxic, leaving a gray area between 10 and 50 ppb. With or without intent, this gray area, along with the associated costs of compliance, led to a proposal to maintain the limit at 50 ppb through a moratorium until an epidemiological study specific to the country's context is conducted. In Argentina, where even the 50 ppb limit is exceeded in some regions (8), this study is yet to be completed after 15 years… In 2021 there was a “regulation agreement” that states the limit of 10 ppb but tolerates up to 50 ppb in “certain conditions” (9). Health loses once again.

## Development of open-source water contaminants detectors as a case study

The initiative to develop biosensors for water contaminants emerged from discussions between a group of makers/entrepreneurs and a group of students/graduates. Both groups were interested in synthetic biology and committed to the social value of applying technology (10). While sharing the common goal of improving people's lives, differing opinions emerged regarding whether to prioritize technology itself or the associated societal value. As this commentary will reveal, both emphases can undermine the intended objective.

From the initial naivety of attempting to decontaminate one of the most polluted water bodies in the Americas (11), the objective quickly shifted to a more realistic goal: detecting specific contaminants in water intended for human consumption. Despite deep conviction and enthusiasm, numerous challenges hindered its progress. Notably, the disconnection between the statements made by companies, regulatory bodies, and funding organizations, and their effective support for possible life-changing innovations, became apparent. Lack of coordination among these entities often hampers the timely resolution of urgent problems.

### Prioritizing the spotlight of an international competition vs. territorial work

The initial quandary revolved around a choice: should we focus on harnessing group's inherent skills and strengths that align with the social issue, or, alternatively, should we prioritize meeting people's demands by utilizing available tools (even if they may not be the optimal for the specific problem at hand). This led to the formation of two working groups, both demonstrating good performance. The Buenos Aires iGEM (12) team 2013 eventually won a prize in a worldwide competition by developing a device to measure arsenic levels (2, 13). Briefly, the device comprised genetically modified bacteria whose color changed in response to the presence of arsenic, and whose intensity corresponded to its concentration. Its design and implementation in an open source domestic device merited the earning of the National Innovation award (14–16) among others. The second group has been conducting fieldwork in several places in Argentina and published the co-development of a biosensor for herbicides (17). However, a critical conflict arose when the group advocating for co-development sought access to resources generated by the iGEM team potentially patentable, leading to tensions and ultimately the separation of the two teams. As in many other instances, personal egos cannot be excluded as a source of conflict. Unfortunately, the synergy in a collaboration is inversely proportional to the collaborators' egos.

### Entrepreneurs and patents

During the development of innovative products in relevant areas, entrepreneurial interests, investors, entrepreneurship competitions, and entrepreneurship promoters inevitably emerge. The first recommendation is often to protect intellectual property and “not publish anything.” This clearly contradicts the initial objective, but may be reluctantly accepted as a means to make the product's development viable and beneficial to the population. Regardless it may be a useful tool, the lack of communication of results and advances threatens the advancement of science and its implementation in practical developments. Especially in institutions that are not very agile and with limited resources. In the case of the University of Buenos Aires it took nearly 1 year to decide on the patentability of the development, ultimately concluding that it was not patentable. This produced an unnecessary delay in the development and making the technology accessible to people.

###  “Development within an academic institution is limited”

Undertaking development within an academic institution poses significant challenges. Resources are limited, and inertia often compels individuals to remain within the academic system, where the promise of security outweighs probable impact in society. In the referred project, one senior and two postdoctoral researchers who expressed interest in commercial development were constrained by the academic system and ultimately discontinued their involvement with the potential product development. After the attempt to transform academics into business professionals proved unsuccessful, we decided to reallocate some resources from basic research to technological development and started offering services through the University. However, establishing a technology-based company is often viewed with suspicion, and only those who have already decided to abandon their academic careers undertake such ventures, which limits the number of people involved. Furthermore, there is a narrative of promoting innovation through converting researchers into entrepreneurs, fuelled by venture capitals. In many cases this is a trap where many researchers fall out of the system and only favor the capital which, eventually, found unicorns. Thus, the capital centered trend is to mine brains or ideas as any other resource.

### “Views from the entrepreneurship perspective”: accessibility vs. profitability

Rather than empowering citizens, the companies prefer to deal with water providers, who are fewer in number and possess greater resources. An economic model that emphasizes affordability, accessibility, open-source solutions without patents, holds little interest for companies. They prefer an exclusive niche market with significant barriers to entry to maximize profits. Notably, it happens that it is easier to control a bunch of water providers than countless empowered people.

### Mechanisms and justifications to avoid the warranty to access clean water are perverse

Some real examples are illustrated below. This section entails delicate anecdotes presented generically without singling out individuals but rather addressing the underlying mechanisms.

“Take it or leave it”: in various conversations with water providers, the alternative of providing either 50 ppb or no arsenic-free water at all was raised, which can be seen as extortion. Additionally, it was common to hear that people are accustomed to consuming such water quality and reject arsenic-free alternatives due to their taste or because they have been consuming this water for generations without apparent harm. Rather than empathy with suffering people, this sounds as a justification of the lack of investment from companies and governments.

“Remineralizing with Arsenic-Contaminated Water”: a peculiar situation arises from one of the treatment methods for arsenic removal: reverse osmosis, which is effective but expensive. Afterward, the purified water requires re-mineralization. Since mineralization through the addition of salts is costly, the purified water is mixed with raw well water. In other words, to save some money, the water is re-mineralized with arsenic-contaminated well water while simultaneously diluting the arsenic concentration with demineralized water.

“Boiling Water Contaminated with Arsenic or Lead”: among the affected population, whether facing lead or arsenic contamination, it is common to hear that they are aware of the problem and thus boil the water before consumption. Needless to say, boiling not only fails to solve the problem but can also worsen it. It is crucial to engage in co-development and consider the target population's mental models to ensure effective solutions.

“Choosing between arsenic and glyphosate”: in some regions of Argentina, the only alternatives seem to be consuming arsenic-contaminated well water or collecting rainwater contaminated with glyphosate (due to extensive application by plane). These are not viable alternatives. The government must intervene to eliminate glyphosate from rainwater and provide means to filter well water or provide bottled water or, better yet, supply safe water through the piping network.

“Withholding Information to Prevent Panic”: when water contamination is suspected, it is common to hear statements, often from decision-making authorities, that it is better to address the problem without alerting the population to prevent panic and potential uncontrolled reactions. In practice, they not only prevent people from panicking, but they don't communicate the problem at all.

“Expecting a recognizing institution to certify absence while ignoring presence”: perhaps the most ethically problematic demand is the explicit request for the University or CONICET (National Scientific and Technical Research Council) to certify the absence of toxic levels of contaminants, while deliberately avoiding knowledge of their presence. This fear of opening Pandora's box leads regulatory authorities to “prefer” not to innovate, as detection of toxins would require them to address contamination issues that are already evident. The argument is that if toxins are detected, tourism and economic activities would need to be suspended, thereby creating tension in which, most of the time, the public health loses. This is true, not only for water providers but also for field producers who don't want their product to be measured for arsenic content to avoid market rebuttal of their goods.

### What the eye doesn't see, the heart doesn't grieve over

In the ongoing dispute between governments unwilling to acknowledge high pollution levels and citizens exaggerating their presence, what is needed is precise and certified measurements over time and across different locations to determine the true extent of contamination. In the absence of reliable measurements from the government, these measurements could come from the Ombudsman's Office, NGOs, or even the community itself. Otherwise, public health is compromised.

## Discussion

In summary, this journey illustrates several tensions between public and commercial interests and how the latter influence government regulatory offices and policies, mostly in pernice of the public (at least, in the short term). However, it also illustrates that there are other tensions and conflicts intrinsic to the scientific-technological systems that adopted a capitalist extractivist model and tend to prompt individualism and title of property rather than collaborative production and social benefit of knowledge.

## Author contributions

AN: Conceptualization, Funding acquisition, Writing—original draft, Writing—review & editing.
